# Phenylephrine preconditioning in embryonic heart H9c2 cells is mediated by up-regulation of SUR2B/Kir6.2: A first evidence for functional role of SUR2B in sarcolemmal K_ATP_ channels and cardioprotection

**DOI:** 10.1016/j.biocel.2015.10.029

**Published:** 2016-01

**Authors:** Sofija Jovanović, Thomas Ballantyne, Qingyou Du, Miloš Blagojević, Aleksandar Jovanović

**Affiliations:** a Medical Research Institute, Division of Cardiovascular & Diabetic Medicine, Ninewells Hospital & Medical School, University of Dundee, UK; b Department of Anatomy, Faculty of Veterinary Medicine, University of Belgrade, Belgrade, Serbia

**Keywords:** SUR2B, Cardioprotection, Preconditioning, Cardiomyocytes, H9c2 cells

## Abstract

ATP-sensitive K^+^ (K_ATP_) channels were originally described in cardiomyocytes, where physiological levels of intracellular ATP keep them in a closed state. Structurally, these channels are composed of pore-forming inward rectifier, Kir6.1 or Kir6.2, and a regulatory, ATP-binding subunit, SUR1, SUR2A or SUR2B. SUR1 and Kir6.2 form pancreatic type of K_ATP_ channels, SUR2A and Kir6.2 form cardiac type of K_ATP_ channels, SUR2B and Kir6.1 form vascular smooth muscle type of K_ATP_ channels. The presence of SUR2B has been described in cardiomyocytes, but its functional significance and role has remained unknown. Pretreatment with phenylephrine (100 nM) for 24 h increased mRNA levels of SUR2B and Kir6.2, without affecting those levels of SUR1, SUR2A and Kir6.1 in embryonic heart H9c2 cells. Such increase was associated with increased K^+^ current through K_ATP_ channels and Kir6.2/SUR2B protein complexes as revealed by whole cell patch clamp electrophysiology and immunoprecipitation/Western blotting respectively. Pretreatment with phenylephrine (100 nM) generated a cellular phenotype that acquired resistance to chemical hypoxia induced by 2,4-dinitrophenol (DNP; 10 mM), which was accompanied by increased in K^+^ current in response to DNP (10 mM). Cytoprotection afforded by phenylephrine (100 nM) was abolished by infection of H9c2 cells with adenovirus containing Kir6.2AFA, a mutant form of Kir6.2 with largely reduced K^+^ conductance. Taking all together, the present findings demonstrate that the activation of α1-adrenoceptors up-regulates SUR2B/Kir6.2 to confer cardioprotection. This is the first account of possible physiological role of SUR2B in cardiomyocytes.

## 1 Introduction

ATP-sensitive K^+^ (K_ATP_) channels were originally described in cardiomyocytes, where physiological levels of intracellular ATP keep them in a closed state. Upon a fall in intracellular ATP concentration, as it happens during ischaemia/hypoxia, the channels are released from inhibition, leading to hyperpolarisation and shortening of action membrane potential to inhibit voltage operated Ca^2+^ channels and prevent intracellular Ca^2+^ overload, which is the main cause of cardiomyocytes death under adverse conditions (reviewed by Nichols, 2006). Structurally, K_ATP_ channels are composed of pore-forming inward rectifier, Kir6.1 or Kir6.2, and a regulatory, ATP-binding subunit, SUR1, SUR2A or SUR2B. It is generally accepted that four Kir6.x and four SURx are physically associated with each other to form functional K_ATP_ channels. The properties of these channels are different in various tissues due to the combinations of the subunits forming the channel. SUR1 and Kir6.2 form pancreatic type of K_ATP_ channels, SUR2A and Kir6.2 form cardiac type of K_ATP_ channels, SUR2B and Kir6.1 form vascular smooth muscle type of K_ATP_ channels and others (Nichols, 2006). There are studies suggesting that further diversity may be generated by combination of more than one type of Kir6.x and SURx subunits within an individual channel (Flagg et al., 2010). All channel subunits have been found in the heart (Du et al., 2006, Zhou et al., 2007, Flagg et al., 2010), but it seems that expression varies from region to region and can change under pathophysiological conditions (Arakel et al., 2014). In the heart, the activation and trafficking of sarcolemmal K_ATP_ channels mediate ischaemic preconditioning, a phenomenon when a brief period of blood vessel occlusion enhances the survival of cardiac cells under conditions that induce myocardial infarction (Budas et al., 2004, Sukhodub et al., 2007).

H9c2 cells are embryonic rat heart cell line that was used with success to study K_ATP_ channels and cardioprotection (reviewed by Jovanović and Jovanović, 2007). These cells express Kir6.1, Kir6.2, SUR1, SUR2A and SUR2B subunits, as adult cardiomyocytes do (Du et al., 2006, Du et al., 2010a, Zhou et al., 2007, Ballantyne et al., 2013). In adult hearts/cardiomyocytes and H9c2 cells, it has been demonstrated that increased SUR2A levels confer cardioprotection by increasing levels of SUR2A-containing K_ATP_ channels (Du et al., 2006, Du et al., 2010a, Du et al., 2010b, Mohammed Abdul et al., 2014, Mohammed Abdul et al., 2015a, Mohammed Abdul et al., 2015b). In contrast, knock-out of SUR1 induces cardioprotection (Lefer et al., 2009). Although SUR2B is expressed in adult cardiomycoytes as well as in H9c2 cells (Du et al., 2006, Du et al., 2010a, Zhou et al., 2007), no functional role for SUR2B-containing K_ATP_ channels has been uncovered. One possible reason why there is no more data about SUR2B in cardiomyocytes could be due to the fact that electrophysiological properties between SUR2A/Kir6.2 and SUR2B/Kir6.2 are indistinguishable (Fujita and Kurachi, 2000) and that it is usual assumption that SUR2A/Kir6.2 is the main sarcolemmal K_ATP_ channel form and, consequently, all K_ATP_ channels-mediated effects are usually ascribed to SUR2A/Kir6.2.

It is well established that pretreatment with α1-adrenoceptor agonist, phenylephrine, induces preconditioning by increasing K_ATP_ channels-mediated current (Turrell et al., 2011). This effect of phenylephrine could be mediated not only by the activation of K_ATP_ channels, but also by their increased presence in plasma membrane, as it has been already described for ischaemic preconditioning (Budas et al., 2004, Sukhodub et al., 2007). In the present study we used H9c2 cells to examine whether pretreatment with phenylephrine would affect the expression of K_ATP_ channels. We found that it would, but not in a way we expected. Phenylephrine increased SUR2B/Kir6.2, without affecting SUR2A levels, to confer cytoprotection. This is the first report ever demonstrating physiological importance and functional role of SUR2B in cardiomyocytes.

## 2 Materials and methods

### 2.1 H9c2 cells

Rat embryonic heart H9c2 cells (ECACC, Salisbury, UK) were cultured in a tissue flask (at 5% CO_2_) containing Dulbecco's modified Eagle's medium supplemented with 10% fetal calf serum and 2 mM glutamine as described (Du et al., 2010a). Briefly, for electrophysiological experiments, the cells were plated on a 35 mm × 10 mm culture dish containing 25-mm glass cover-slips. The cells were cultured in incubators (Galaxy, oxygen control model, RS Biotech, Irvine, UK). Some cells were treated with phenylephrine (100 nM) or vehicle (methanol) for 24 h. For some experiments H9c2 cells were infected with adenoviral constructs containing either luciferase (cells infected with luciferase have served as control cells in this study) or Kir6.2AFA (a mutant form of Kir6.2 where the pore GFG was mutated into AFA leading to largely reduced K^+^ conductance).

### 2.2 Real time RT-PCR

Real time RT-PCR was performed as described previously (Du et al., 2006, Du et al., 2010a, Du et al., 2010b). In brief, total RNA was extracted from H9c2 cells using TRIZOL reagent (Invitrogen, Carlsbad, CA) according to the manufacturer's recommendations. Extracted RNA was further purified by RNeasy Plus Mini Kit (Qiagen, Crawley, UK) according to the manufacturer's instructions. The primers had the following sequences. For SUR1 sense – 5′-GGAAGGACTCACCCCATC-3′ and for antisense – 5′-GAGACCATCAAGGCATA GG-3′. For SUR2A sense – 5′-ACTTCAGCGTTGGACAGAGAC-3′ and for antisense – 5′-AGCAAGGTTTGGACCAGTATCG-3′. For SUR2B sense – 5′-GACGCCACT GTCACCGAAG-3′ and for antisense – 5′-TCATCACAATGACCA GGTCAGC-3′. For Kir6.1 sense – 5′-GTCACACGCTGGTCATCTTCAC-3′ and for antisense – 5′-GGCACTCCTCAGTCATCATTCTCC-3′. For Kir6.2 sense – 5′-TGGCTGACGAGATTCTGTGG-3′ and for antisense – 5′-TGGCGGGCTG TGCAGAG-3′. Glyceraldehyde 3-phosphate dehydrogenase (GAPDH) was used as a control and for GAPDH sense – 5′-ATA GAATTCC ATGACAAAGTGGACATTGTTGCCA-3′ and for antisense – 5′-AGCCT CGAGTTAGGAAATGAG CTTCACAAAGTT-3′. The reverse transcription (RT) reaction was carried out with ImProm-II Reverse Transcriptase (Promega, Southampton, UK). A final volume of 20 μl of RT reaction containing 4 μl of 5× buffer, 3 mM MgCl_2_, 20 U of RNasin^®^ Ribonuclease inhibitor, 1 U of ImProm-II reverse transcriptase, 0.5 mM each of dATP, dCTP, dGTP, and dTTP, 0.5 μg of oligo(dT), and 1 μg of RNA was incubated at 42 °C for 1 h and then inactivated at 70 °C for 15 min. The resulting cDNA was used as a template for real-time PCR. A SYBR Green I system was used for the RT-PCR and the 25 μl reaction mixture contained: 12.5 μl of iQ™ SYBR^®^ Green Supermix (2×), 7.5 nM each primers, 9 μl of ddH_2_O, and 2 μl of cDNA. In principle, the thermal cycling conditions were as follows: an initial denaturation at 95 °C for 3 min, followed by 40 cycles of 10 s of denaturing at 95 °C, 15 s of annealing at 56 °C, and 30 s of extension at 72 °C. The real-time PCR was performed in the same wells of a 96-well plate in the iCycler iQ™ Multicolor Real-Time Detection System (Bio-Rad, Hercules, CA). Data was collected following each cycle and displayed graphically (iCycler iQ™ Real-time Detection System Software, version 3.0A, BioRad, Hercules, CA). Primers were tested for their ability to produce no signal in negative controls by dimer formation and then with regard to the efficiency of the PCR. Efficiency is evaluated by the slope of the regression curve obtained with several dilutions of the cDNA template. Melting curve analysis tested the specificity of primers. Threshold cycle values, PCR efficiency (examined by serially diluting the template cDNA and performing PCR under these conditions) and PCR specificity (by constructing the melting curve) were determined by the same software, as described in Du et al. (2010a). The calculation of relative mRNA expression was performed as described (Sudhir et al., 2011). The relative expression ratio (*R*) of gene encoding channel subunits is calculated using equation *R* = (*E*_K_)^ΔCP_K_(C-P)^/(*E*_R_)^ΔCP_R_(C-P)^, where *E*_K_ is the real time PCR efficiency of a gene of interest transcript, *E*_R_ is the real time PCR efficiency of a reference gene (GAPDH; we have shown that this gene is not affected by phenylephrine), ΔCP_K_ is the crossing point deviation of control (C)–phenylephrine (P) of gene of interest transcript while ΔCP_R_ is the crossing point deviation of control (C)–phenylephrine (P) of a reference gene transcript. All primers and conditions applied in the present study have been fully validated previously (Du et al., 2010a, Sudhir et al., 2011).

### 2.3 Patch clamp electrophysiology

To monitor whole cell K^+^ current the giagaohm seal patch-clamp technique was applied in the traditional or perforated-patch whole cell configuration. For whole-cell electrophysiology cells were superfused with Tyrode solution (in mM: 136.5 NaCl; 5.4 KCl; 1.8 CaCl_2_; 0.53 MgCl_2_; 5.5 glucose; 5.5 HEPES–NaOH; pH 7.4). Pipettes (resistance 3–5 MΩ) were filled with (in mM): KCl 140, MgCl_2_ 1, ATP 0.005 (low ATP was present to prevent run-down of the channel without significantly affecting channel activity), HEPES–KOH 5 (pH 7.3). For perforated patch clamp electrophysiology, the same solutions were used just ATP was completely omitted and amphotericin B (Sigma, 240 μg/ml) added (Du et al., 2010a). For all cells monitored, the membrane potential was normally held at −40 mV and the currents evoked by a series of 400 ms depolarising and hyperpolarising current steps (−40 mV to +80 mV in 20 mV steps) recorded directly to hard disk using anAxopatch-200B amplifier, Digidata-1321 interface and pClamp8 software (Axon Instruments, Inc., Forster City, CA). The capacitance compensation was adjusted to null the additional whole-cell capacitative current. The slow capacitance component measured by this procedure was used as an approximation of the cell surface area and allowed normalisation of current amplitude (i.e. current density). Currents were low pass filtered at 2 kHz and sampled at 100 μs intervals, as described in Du et al. (2010a).

### 2.4 Immunoprecipitation/Western blotting

Immunoprecipitation/Western blotting was performed as previously described (Du et al., 2006, Du et al., 2010a). To obtain cellular membrane fraction cells were homogenised in buffer I (Tris 10 mM, NaH_2_PO_4_ 20 mM, EDTA 1 mM, PMSF 0.1 mM, pepstatin 10 μg/ml, leupeptin 10 μg/ml, at pH = 7.8) and incubated for 20 min (at 4 °C). The osmolarity was restored with KCl, NaCl and sucrose and the obtained mixture was centrifugated at 500 × *g*. The supernatant was diluted in buffer II (imidazole 30 mM, KCl 120 mM, NaCl 30 mM, NaH_2_PO_4_ 20 mM, sucrose 250 mM, pepstatin 10 μg/ml, leupeptin 10 μg/ml, at pH = 6.8) and centrifugated at 7000 × *g*, pellet removed and supernatant centrifugated at 30,000 × *g*. The obtained pellet contains membrane fraction. Protein concentration was determined using the method of Bradford; 10 μg of the anti-Kir6.2 antibody was prebound to Protein-G Sepharose beads and used to immunoprecipitate from 50 μg of membrane fraction protein extract. The pellets of this precipitation were run on SDS-polyacrylamide gels for Western analysis. Western blot probing was performed using 1/1000 dilution of anti-SUR2B antibody, and detection was achieved using Protein-G HRP and ECL reagents. The band intensities were analyzed using the Quantiscan software.

### 2.5 Cell survival assay

The survival of H9c2 cells was assayed using Multitox-Fluor Multiplex Cytotoxicity Assay (Promega). Briefly, H9c2 cells were plated in complete media (DMEM containing 10% FCS) in 96-well plate. After incubation period (24 h), the DNP was added to each well at the final concentration of 10 mM. To measure cell survival 6 h later, the peptide substrate (GF-AFC) that can be cleaved only by live cells was added to the each well. Following 30 min-long incubation at 37 °C, plates were measured using 1420 Multibabel Counter (Victor) plate reader, with excitation at 370 nm and emissions of 480 nm. The percentage of live cells was calculated based on the intensity of fluorescence according to the manufacturer instructions, as described in Du et al. (2010a).

### 2.6 Statistical analysis

Data are presented as mean ± SEM, with n representing the number of cells (for electrophysiology) or independent experiments (for real time RT-PCR and cell survival assay). Mean values were compared by Student's *t*-test using SigmaStat program (Jandel Scientific, Chicago, Illinois). *P* < 0.05 was considered statistically significant.

## 3 Results

### 3.1 Pretreatment with phenylephrine increases SUR2B/Kir6.2

**Fig. 1:**
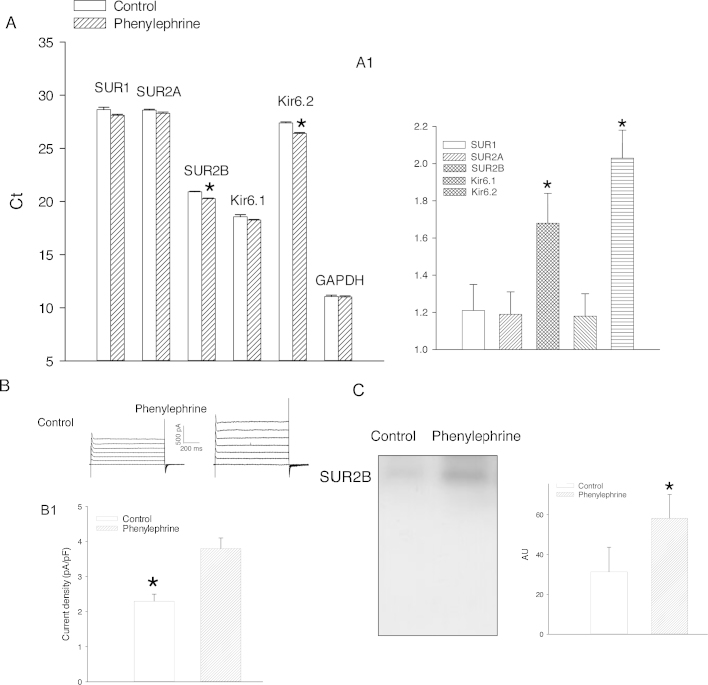
Pretreatment with phenylephrine up-regulate SUR2B/Kir6.2 leading to increase in number of functional K_ATP_ channels. (A) Bar graphs representing cycling threshold of the real time RT-PCR progress curves of K_ATP_ channel-forming subunits. Each bar represents mean ± SEM (*n* = 6). *P < 0.05. (A1) Relative expression ratio (control/phenylephrine ratio of mRNA levels calculated from data shown in A) of K_ATP_ channel subunits (as depicted on the graph). Each bar represents mean ± SEM (*n* = 6). (B) Original whole cell membrane currents for cells kept under control conditions (control) and pretreated with phenylephrine (100 nM). (B1) Bar graphs of K^+^ current density at +80 mV under conditions corresponding to (B). Each bar represents mean ± SEM (*n* = 5). *P < 0.05. (C) Original Western blots with anti-SUR2B antibody of anti-Kir6.2 immunoprecipitate pellets from membrane fractions obtained from H9c2 cells under control conditions and when pretreated with phenylephrine (100 nM) and corresponding graph. Each bar represents mean ± SEM (*n* = 3–4). *P < 0.05.

24 h-long treatment of H9c2 cells with phenylephrine (100 nM) did not affect mRNA levels of SUR1 (cycling threshold was 28.65 ± 0.22 for control and 28.12 ± 0.13 for phenylephrine-pretreated cells, *P* = 0.069; *n* = 6; Fig. 1AA1), SUR2A (cycling threshold was 28.60 ± 0.09 for control and 28.12 ± 0.15 for phenylephrine-pretreated cells, *P* = 0.131; Fig. 1AA1) and Kir6.1 (cycling threshold was 18.56 ± 0.21 for control and 18.25 ± 0.06 for phenylephrine-pretreated cells, *P* = 0.209; *n* = 6; Fig. 1AA1). On the other hand, this treatment significantly increased levels of SUR2B and Kir6.2 mRNAs (SUR2B: cycling threshold was 20.92 ± 0.05 for control and 20.28 ± 0.05 for phenylephrine-pretreated cells, *P* < 0.001; *n* = 6; Fig. 1AA1; Kir6.2: cycling threshold was 27.40 ± 0.10 for control and 26.40 ± 0.10 for phenylephrine-pretreated cells, *P* < 0.001; *n* = 6; Fig. 1AA1). The level of glyceraldehyde 3-phosphate dehydrogenase (GAPDH) that we used as a housekeeping gene was not affected by 100 nM phenylephrine (cycling threshold was 11.05 ± 0.14 for control and 11.01 ± 0.11 for phenylephrine-pretreated cells, *P* = 0.601; *n* = 6; Fig. 1A). In H9c2 cells pretreated with phenylephrine (100 nM), K^+^ current density was significantly increased (current density at +80 mV was 2.3 ± 0.2 pApF for control and 3.8 ± 0.3 pApF for phenylephrine-pretreated cells; *P* < 0.001; *n* = 5; Fig. 1BB1). Western blotting signal obtained by anti-SUR2B antibody on anti-Kir6.2 immunoprecipitate was significantly stronger in H9c2 cells treated with phenylephrine (100 nM) than in untreated ones (Fig. 1C).

### 3.2 Pretreatment with phenylephrine improves the outcome of chemical hypoxia

**Fig. 2:**
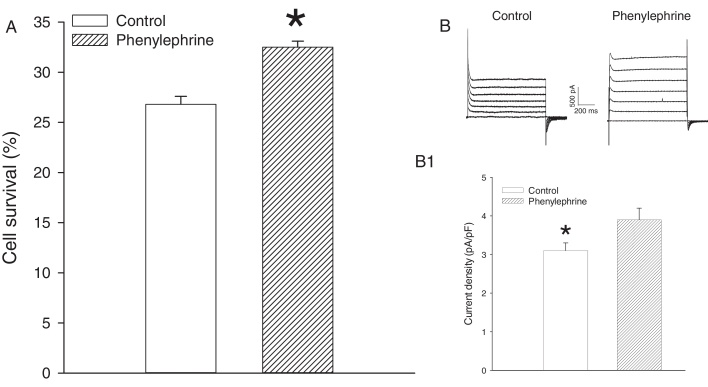
Pretreatment with phenylephrine increases survival of cells and whole cell K^+^ current in response to DNP. (A) A bar graph showing a percentage of survival in control cells and cells pretreated with phenylephrine (100 nM) exposed to DNP (10 mM). Each bar represent mean ± SEM (*n* = 6). *P < 0.05. (B) Original perforated patch clamp whole cell membrane currents in response to DNP (10 mM) for cells kept under control conditions (control) and pretreated with phenylephrine (100 nM). (B1) Bar graphs of K^+^ current density at +80 mV under conditions corresponding to (B). Each point represents mean ± SEM (*n* = 6). *P < 0.05.

To determine whether increased number of functional SUR2B/Kir6.2 channels alters in any way cellular resistance to stress, we have used DNP, an inhibitor of oxidative phosphorylation. 26.8 ± 0.8% of control cells survived challenge with DNP (10 mM; Fig. 2A) which was significantly lower than those in cells pretreated with 100 nM phenylephrine (32.5 ± 0.6% cells survived challenge with DNP, *P* < 0.001, *n* = 6, Fig. 2A). At the same time, DNP (10 mM) induced significantly larger K+ current in cells pretreated with phenylephrine (100 nM) than those in control cells (current density at +80 mV was 3.1 ± 0.2 pApF for control and 3.9 ± 0.3 pApF for phenylephrine-pretreated cells; *P* < 0.001; *n* = 6; Fig. 2BB1).

### 3.3 Kir6.2AFA inhibits cytoprotection afforded by phenylephrine

**Fig. 3:**
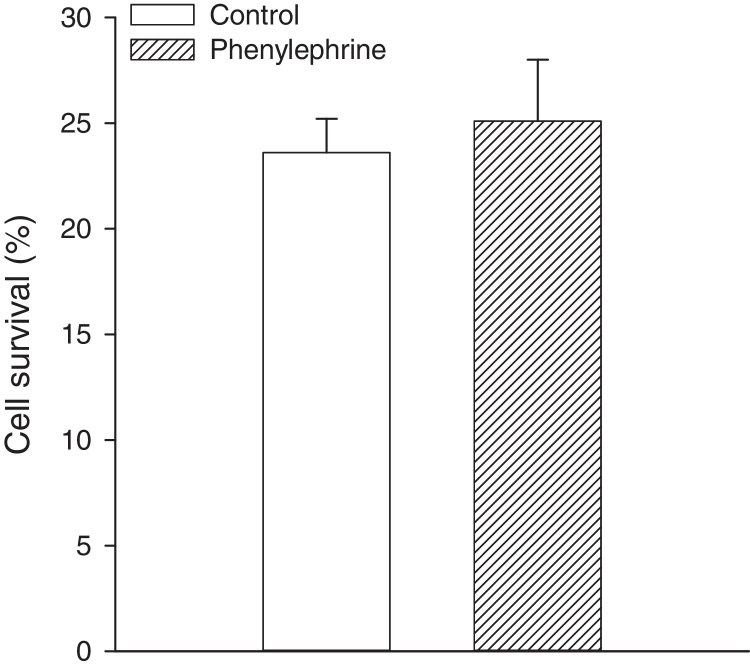
Kir6.2AFA inhibits cytoprotection afforded by phenylephrine. A bar graph showing a percentage of survival in cells infected with Kir6.2AFA kept under control conditions and pretreated with phenylephrine (100 nM) exposed to DNP (10 mM). Each bar represent mean ± SEM (*n* = 6).

To establish a causal relationship between channels opening and cytoprotection afforded by phenylephrine, we have tested the effect of Kir6.2AFA. This is a mutant form of Kir6.2 with largely reduced K^+^ conductivity (Du et al., 2010a). We have found out that H9c2 cells expressing Kir6.2AFA cannot be preconditioned by phenylephrine as cell survival was 25.1 ± 2.9%, which was not significantly different from those in control cells (cell survival was 23.6 ± 1.6%, *P* = 0.611, *n* = 6; Fig. 3).

## 4 Discussion

H9c2 cells are well-established experimental model that is similar to adult cardiomyocytes in crucial aspects of K_ATP_ channels structure, physiology and function. It has been shown that both cell types express all five K_ATP_ channel subunits (Du et al., 2006, Du et al., 2010a, Zhou et al., 2007) and that increased levels of SUR2A increase numbers of fully functional K_ATP_ channels resulting in a phenotype more resistant to stress (Du et al., 2006, Du et al., 2010a). Signalling pathways regulating K_ATP_ channel levels and mediating preconditioning and cardioprotection are similar between adult cardiomyocytes and H9c2 cells (Ranki et al., 2002, Crawford et al., 2003, Qu et al., 2010, Du et al., 2010a, Du et al., 2010b, Mohammed Abdul et al., 2015a, Mohammed Abdul et al., 2015b, Kuznetsov et al., 2015). Both cell types respond similarly to an inhibitor of oxidative phosphorylation, DNP (Jovanović et al., 1998, Jovanović et al., 2009a, Jovanović et al., 2009b, Jovanović and Jovanović, 2001a, Du et al., 2010a), which seems to be a good experimental model to study myocardial response to ischaemia/ischaemia-reperfusion and cardioprotection (Jovanović and Jovanović, 2007). SUR2A and SUR2B are splicing variants of the same ABCC9 gene (Nichols, 2006, Flagg et al., 2010). While SUR2A is the main cardiac responder to a range of hormones and physiological stimuli including estrogens, physical activity, ageing and oxygen, no cardiac role/function has been ascribed for SUR2B, a protein that is recognized mainly as a regulatory subunit of vascular type of K_ATP_ channels (Nichols, 2006, Flagg et al., 2010). Cardiac preconditioning afforded by α1-adrenoceptor agonist is mediated via AMPK (Turrell et al., 2011), which is known to increase the number of sarcolemmal K_ATP_ channels (Sukhodub et al., 2007). As the rate limiting factor in functional channel formation is availability of SUR2A (Du et al., 2006, Sukhodub et al., 2010, Sukhodub et al., 2011), we have expected that phenylephrine could up-regulate SUR2A. However, that was not the case and phenylephrine did not affect expression of SUR1, SUR2A and Kir6.1. On the other hand, phenylephrine increased mRNA levels of SUR2B and Kir6.2. SUR2B and Kir6.2 can physically associate to form K_ATP_ channels (Fujita and Kurachi, 2000). In the heart, SUR2B has been primarily localized in subcellular structures, mitochondrias and endoplasmic reticulum, although presence in the sarcolemma was recognised as well (Zhou et al., 2007). If increased SUR2B/Kir6.2 results in more functional K_ATP_ channels in sarcolemma, patch clamp electrophysiology should detect such change (Du et al., 2010b). K_ATP_ channels are normally closed by millimolar intracellular ATP (Nichols, 2006). To measure K^+^ current flowing through K_ATP_ channels we used traditional whole cell patch clamp electrophysiology with small presence of ATP in the pipette solution. Used amounts of ATP were not sufficient to significantly inhibit channels opening, but helped to keep channels in operative condition and prevent channels run-down (Jovanović and Jovanović, 2005). These measurements demonstrated that cells pretreated with phenylephrine exhibit increase in K^+^ current flowing through K_ATP_ channels, which supports the notion that up-regulation of SUR2B/Kir6.2 results in increased number of functional channels in sarcolemma. Finally, we have tested whether pretreatment with phenylephrine would increase the levels of SUR2B that physically associate with Kir6.2. To do that, we have applied immunoprecipitation with anti-Kir6.2 antibody followed by Western blotting, with anti-SUR2B antibody. This strategy secures exclusive measurement of Kir6.2 and SUR2B that physically interact with each other to form functional channels (Ranki et al., 2001, Du et al., 2006, Du et al., 2010a, Sukhodub et al., 2010, Sukhodub et al., 2011). Significantly stronger western blot signal was obtained in cells pre-treated with phenylephrine suggesting that phenylephrine increased the level of SUR2B/Kir6.2 in membrane of H9c2 cells. Thus, findings obtained by patch clamp electrophysiology and immunoprecipitation/Western blotting support each other and provide strong evidence in favor of the notion that phenylephrine increases the number of functional SUR2B/Kir6.2 K_ATP_ channels. This is the first demonstration of regulation of SUR2B-containing K_ATP_ channels in heart cells.

DNP is an inhibitor of oxidative phosphorylation that is routinely used to mimic ischaemia and ischaemia-reperfusion injury and it is often used to study cardioprotection (Jovanović and Jovanović, 2007). We have found that phenylephrine-pretreated cells were more resistant to DNP than the control ones, which was associated with increased K^+^ current in response to DNP. An increase in number of functional K_ATP_ channels is known to increase DNP-induced K^+^ current mediating cytoprotection afforded by K_ATP_ channels (Du et al., 2010a). Thus, the observed effect of DNP further confirms that treatment with phenylephrine increases the number of functional channels that, in turn, mediate cytoprotection. Our current findings are also in accord with a previous study demonstrating that preconditioning afforded by phenylephrine was associated with increased K_ATP_ channels-mediated K^+^ current (Turrell et al., 2011).

It is well established that activation of K_ATP_ channels is cytoprotective even in cells that do not generate action membrane potential, including H9c2 cells (Du et al., 2010a). Activation of K_ATP_ channels hyperpolarize the membrane which, in turn, prevents influx of Ca^2+^ and consequent Ca^2+^ loading, which is the main cause of cell death in stress (Jovanović and Jovanović, 2001a, Jovanović and Jovanović, 2001b). An increase in number of K_ATP_ channels is associated with increased K^+^ current in response to challenge with DNP, which can be inhibited by Kir6.2AFA, a mutant form of Kir6.2 of largely reduced K^+^ conductivity (Jovanović et al., 2009a, Jovanović et al., 2009b, Du et al., 2010a). We have found out that H9c2 cells expressing Kir6.2AFA cannot be preconditioned by phenylephrine, which strongly supports the notion that the observed cardioprotection is mediated by increase in K_ATP_ channels.

SUR2B is a subunit found primarily in smooth muscle cells, although their presence was reported in other cell types as well (Nichols, 2006, Flagg et al., 2010, Du et al., 2010b). In cardiomyocytes, their presence was detected, but functional role was not understood. Here, we have found that SUR2B physically associate with Kir6.2 to act as a regulatory subunit in sarcolemmal K_ATP_ channels to confer cardioprotection. What would be a significance of SUR2B-containing K_ATP_ channels in cardiomyocytes? There is not much difference in electrophysiological properties between SUR2A/Kir6.2 and SUR2B/Kir6.2, but there are some differences in sensitivity towards certain ligands. As an example, in the presence of Mg^2+^ SUR2B/Kir6.2 is slightly more sensitive to intracellular ATP, while in the absence of Mg^2+^ is otherway around. It is also known that SUR2B/Kir6.2 can be activated by diazoxide, which is not the case with SUR2A/Kir6.2 (Fujita and Kurachi, 2000). In addition to that, it is known that SUR2A/Kir6.2 physically associate with adenylate kinase, creatine kinase and glycolytic enzymes *in vivo* (Carrasco et al., 2001, Crawford et al., 2002a, Crawford et al., 2002b, Jovanović et al., 2005, Hong et al., 2011). These enzymes are crucial for the channel regulation during ischaemia/hypoxia and for ATP production, which also seems to contribute to cytoprotection (Jovanović et al., 2009a, Jovanović et al., 2009b). Whether SUR2B/Kir6.2 protein complex interact with the same enzymes as SUR2A/Kir6.2 do it is yet to be determined, but some differences are probable. Consequently, up-regulation of SUR2B/Kir6.2 would have different physiological outcome from up-regulation of SUR2A/Kir6.2. It seems that the activation of α1-adrenoceptors creates a unique cardiac phenotype in terms of K_ATP_ channels expression as other factors such as hypoxia, exercise, estrogens and ageing affect mainly SUR2A-containing K_ATP_ channels.

Taking all together, the present findings demonstrate that activation of α1-adrenoceptors up-regulates SUR2B/Kir6.2 to confer cardioprotection. This is the first account of possible physiological role of SUR2B in cardiomyocytes.
